# Supplementary material for: Saccharomyces boulardii (CNCM I-745) improves intestinal damage in sepsis by remodeling intestinal flora structure
Source: Front Cell Infect Microbiol. 2025 Jul 31;15:1602792. doi: 10.3389/fcimb.2025.1602792 (PMC12350329; doi:10.3389/fcimb.2025.1602792)
Supplement: Supplementary file 1 [file DataSheet1.zip › summary/summary/8_Advanced_analyse/2_PICRUSt2_result/KEGG/Group.CW4_vs_CW0/PICRUSt2_KEGG_level2_Group.CW4_vs_CW0_diff.pdf]

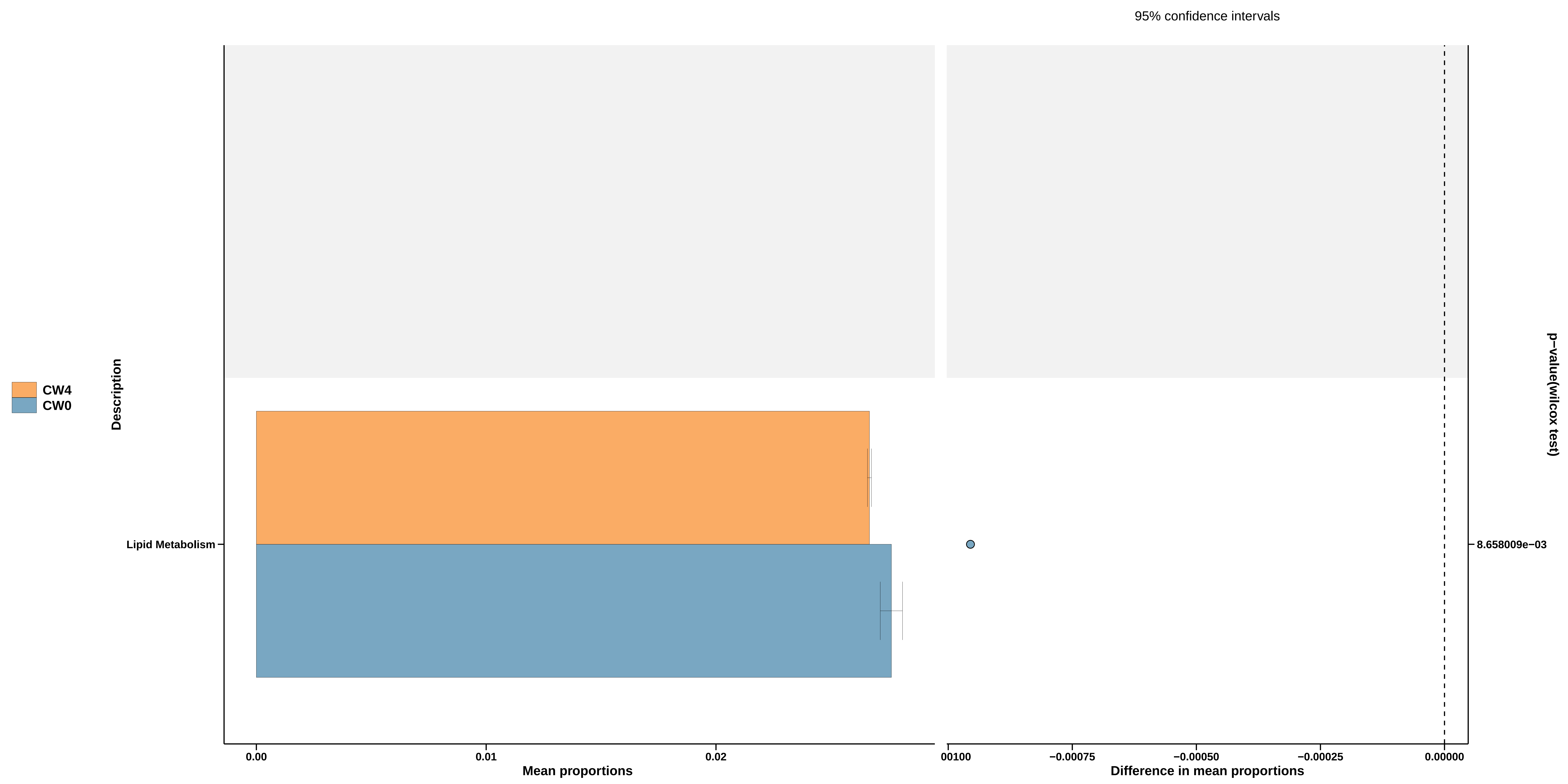
95% confidence intervals
CW4
CW0
Description
Lipid Metabolism
0.00
0.01
0.02
Mean proportions
00100
−0.00075
−0.00050
−0.00025
0.00000
Difference in mean proportions
8.658009e−03
p−value(wilcox test)
